# NAT1 inhibits liver metastasis of colorectal cancer by regulating EMT and glycolysis

**DOI:** 10.18632/aging.205957

**Published:** 2024-06-24

**Authors:** Wang Gu, Chen Li, Tingting Shen, Li Tong, Wenkang Yuan, Xiaofei Zheng, Tianqi Wang, Siyu Wang, Benshuai Zhu, Chong Zhang, Chao Zhang

**Affiliations:** 1 Department of General Surgery, The First Affiliated Hospital of Anhui Medical University, Hefei, Anhui 230022, China; 2 Department of Biology, Chemistry, Pharmacy, Free University of Berlin, Berlin 14195, Germany; 3 Department of Pathology, The First Affiliated Hospital of Anhui Medical University, Hefei, Anhui 230022, China

**Keywords:** NAT1, colorectal cancer (CRC), liver metastasis, epithelial-mesenchymal transition (EMT), glycolysis, VEGF

## Abstract

Metastasis is the primary cause of cancer-related deaths, and colorectal cancer (CRC) liver metastasis is a major poor prognostic factor in CRC. NAT1 (N-acetyltransferase 1) plays a crucial role in the invasive and metastatic processes of colorectal cancer. The role and molecular mechanism of NAT1 on tumor cells were verified by establishing a cell model of overexpression and knockdown of NAT1, and further verified by establishing a liver metastasis model of colorectal cancer for animal experiments. *In vivo* and *in vitro* experiments have demonstrated that overexpression of NAT1 reduces the ability of metastasis and invasion of colorectal cancer cells. NAT1 overexpression inhibits the PI3K/AKT/mTOR signaling pathway, thereby suppressing the EMT (epithelial-mesenchymal transition) process and glycolytic ability of tumor cells. Additionally, decreased glycolytic ability results in reduced VEGF (Vascular endothelial growth factor) expression in colorectal cancer cells. The decreased VEGF expression leads to decreased angiogenesis and vascular permeability in liver metastases, ultimately reducing the occurrence of liver metastasis. Our findings highlight that overexpression of NAT1 significantly inhibits the PI3K/AKT/mTOR signaling pathway, thereby suppressing EMT, glycolytic ability, and VEGF expression in colorectal cancer cells, collectively preventing the development of liver metastasis.

## INTRODUCTION

Colorectal cancer (CRC) is the third most common cancer worldwide and a major contributor to cancer-related mortality [1]. While the 5-year relative survival rate for non-metastatic CRC exceeds 90%, it drops to just over 10% for patients with distal tumor spread [2]. The majority (90%) of colorectal cancer-related deaths are attributed to distal invasion and metastasis [3]. Among distant metastases, hepatic metastasis is the most common site, significantly contributing to the high mortality rate in CRC patients [4]. Surgical resection is currently the most effective treatment for hepatic metastasis; however, approximately 70% of patients will experience tumor recurrence [5]. Moreover, liver metastasis in colorectal cancer is a complex, multi-step process involving complex interactions between the host and malignant cells. Therefore, revealing the molecular mechanism of colorectal cancer progression is of great significance and may provide new and effective anti-tumor strategies for patients with metastatic colorectal cancer.

Epithelial-to-Mesenchymal Transition (EMT) is a crucial process in various developmental events and is considered to be a marker of advanced tumor invasion and metastasis [6, 7]. EMT involves the loss of intercellular adhesion and the acquisition of migratory and invasive properties [8]. It usually occurs during the initial stages of cancer metastasis, when epithelial cells shed the constraints of adhesion molecules such as E-cadherin. EMT and its associated molecules have emerged as potential targets for cancer prognosis and therapy. In the context of colorectal cancer, EMT plays a significant role in liver metastasis. However, our understanding of the precise mechanisms underlying EMT-driven metastasis remains limited. The mechanism by which changing metabolic patterns promotes cell proliferation and growth not only helps cells resist external stress, but also gives cells new functions, a phenomenon known as “metabolic reprogramming.” One of the most prominent examples of this reprogramming is the enhancement of glycolysis, characterized by increased glucose uptake and lactic acid production, originally known as the “Warburg effect response” [9]. This metabolic change is essential for survival and growth of cancer cells as it provides tumor cells with energy, macromolecular precursors, and reducing equivalents [10]. Among the key angiogenesis factors, VEGF (Vascular endothelial growth factor) stands out as being closely associated with the initial stages of tumor development, progression, and metastasis [11]. During angiogenesis, endothelial cells (ECs) must adapt their metabolism to meet the heightened energy and biomass demands associated with their transition from a quiescent state to rapid growth and division [12]. Numerous studies have demonstrated that up to 85% of adenosine triphosphate (ATP) production in ECs relies on aerobic glycolysis rather than oxidative phosphorylation, particularly during angiogenesis, as glycolysis produces ATP at a faster rate compared to oxidative metabolism. This metabolic preference enables ECs to meet their energetic requirements during their dynamic growth and division phases [13, 14]. Recent investigations have unveiled a twofold increase in the rate of glycolysis as stationary endothelial cells transition into rapid vascular sprouting [13].

N-acetyltransferase (NAT) is an enzyme that catalyzes the transfer of an acetyl group from acetyl-CoA to a substrate molecule. This process is called acetylation and plays a crucial role in various metabolic pathways in organisms. There are two main isoforms of human NAT, NAT1 and NAT2, which are encoded by different genes. These enzymes are found primarily in the liver but are also found in other tissues. NAT1 gene is located near chromosome 8p22, showing genetic polymorphism. Numerous studies have confirmed a strong link between NAT1 and tumorigenesis [15–17]. Apart from its involvement in biological transformations, NAT1 has been implicated in the growth and invasion of cancer cells [18, 19]. Specifically, it is up-regulated in estrogen receptor-positive breast cancer [20, 21], while being underexpressed in cervical, prostate, and colorectal cancer [15, 22]. Loss of NAT1 function has been linked to growth inhibition and enhanced expression of E-cadherin protein in colorectal adenocarcinoma [23]. Consequently, NAT1 holds significant research value across various cancer types, necessitating further exploration of its underlying mechanisms. However, the comprehensive role and mechanism of NAT1 in colorectal metabolism remain elusive. NAT1 plays an important role in various cancers, but there are few studies on the specific mechanism of NAT1 in colorectal cancer, especially in the mechanism of liver metastasis in colorectal cancer. Our study will further clarify the role of NAT1 in colorectal cancer and how to regulate the occurrence of liver metastasis in colorectal cancer. All these provide new directions and ideas for the study of liver metastasis mechanism of colorectal cancer. Therefore, the objective of this study is to investigate the impact of NAT1 on colorectal cancer metabolism and provide novel therapeutic insights and targets for the management of colorectal cancer.

## RESULTS

### Low expression of NAT1 in colorectal cancer and liver metastasis patients

**Figure 1:**
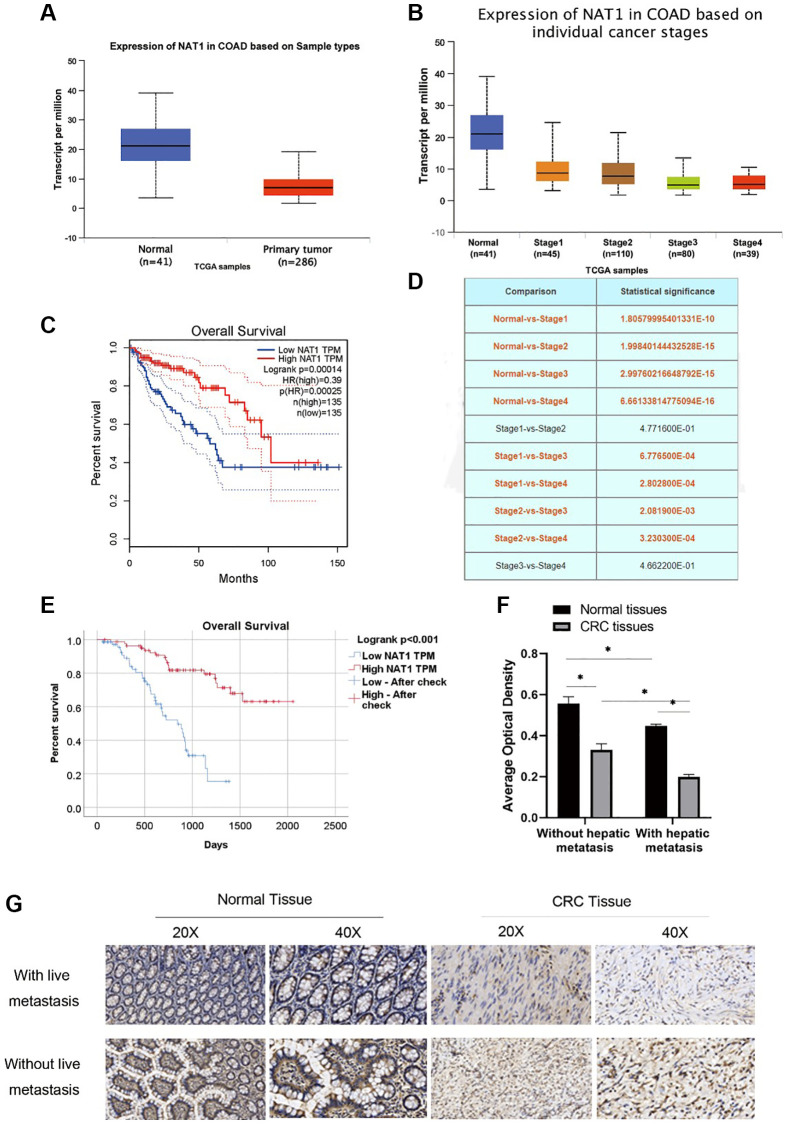
**NAT1 has low expression in patients with colorectal cancer and patients with liver metastasis.** (**A**) TCGA database showed that the expression level of NAT1 was low in patients with colorectal cancer; (**B**) The expression of NAT1 was lower in colorectal cancer patients with higher stage; (**C**) Overall survival was longer in patients with high NAT1 expression; (**D**) NAT1 was statistically different in different stages of colorectal cancer; (**E**) Immunohistochemical results of tissue chips showed that patients with higher NAT1 expression had longer survival; (**F**, **G**) Immunohistochemical results showed that the level of NAT1 in colon cancer tissues of patients with liver metastasis was lower (*P* < 0.05).

NAT1 in the tissue of colorectal expression differences can be found in UALCAN website (http://ualcan.path.uab.edu/index.html). The expression of NAT1 is low in cancer tissues (Figure 1A), and there are differences in the expression of NAT1 in different stages of colorectal cancer. The expression of NAT1 is also different in different stages of colorectal cancer, and the higher the stage, the lower the expression of NAT1. Figure 1D shows the statistical results of the differential expression of NAT1 in different stages (Figure 1B, 1D). Compared with cancer tissue, NAT1 was highly expressed in normal tissue. The survival analysis results showed that patients with high NAT1 expression had longer overall survival (Figure 1C). According to the immunohistochemical results of tissue chips in 155 patients with liver metastasis of colorectal cancer, the patients were divided into high expression group and low expression group for survival analysis. The results showed that the survival of patients in the high expression group was longer (45.23 ± 2.31 months) than that in the low expression group (26.93 ± 1.81 months) (Figure 1E). We also obtained pathological sections of 6 patients with colorectal cancer and 6 patients with liver metastases of colorectal cancer from the Department of Pathology for immunohistochemical experiments. Experimental results showed that, compared with paracancer tissues, NAT1 expression was lower in colorectal cancer patients and cancer tissues of patients with liver metastasis; in addition, the expression of NAT1 in colorectal cancer tissues of patients with liver metastasis was lower than that in cancer tissues of patients without liver metastasis (Figure 1F, 1G), indicating that low NAT1 expression may be related to liver metastasis of colorectal cancer. Low expression of NAT1 may promote liver metastasis of colorectal cancer. Therefore, our study highlights the importance of NAT1 in liver metastasis of colorectal cancer and suggests that NAT1 can be used as a potential prognostic marker and therapeutic target to improve the clinical treatment of metastatic colorectal cancer.

### Overexpression of NAT1 inhibits proliferation, migration and liver metastasis of colorectal cancer cells

**Figure 2:**
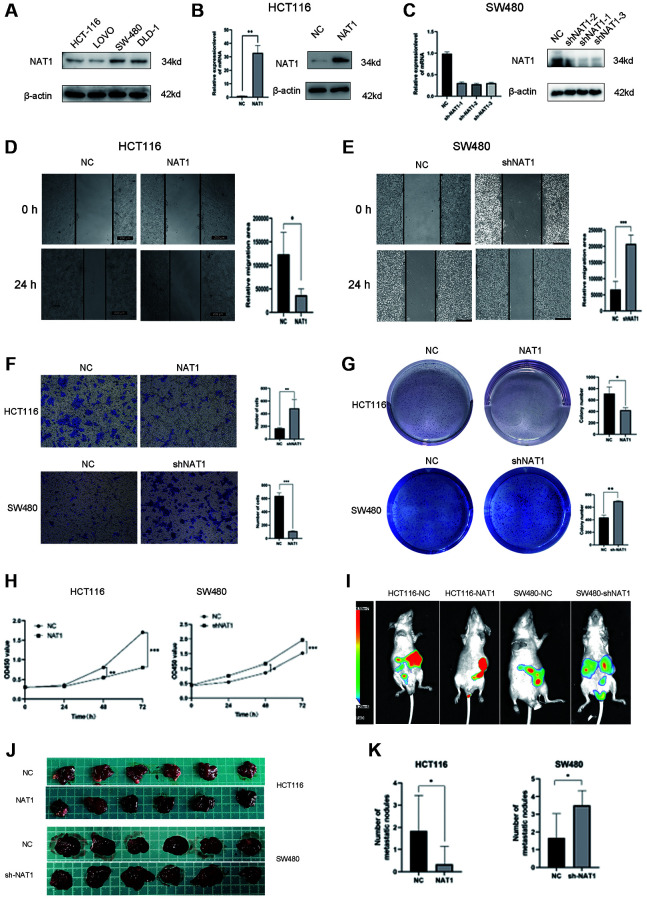
**Overexpression of NAT1 inhibits proliferation, migration and liver metastasis of colorectal cancer cells.** (**A**) NAT1 was highly expressed in HCT116 cell line and low expressed in SW480 cell line; (**B**, **C**) NAT1 was knocked down in HCT116 cell line and was overexpressed in SW480 cell line; (**D**, **E**) Scratch test showed that overexpression of NAT1 could inhibit cell migration ability (*P* < 0.05); (**F**) Transwell assay showed that overexpression of NAT1 significantly inhibited cell invasion (*P* < 0.05); (**G**) Plate cloning results showed that high expression of NAT1 had inhibitory effect on cell proliferation (*P* < 0.05); (**H**) CCK8 assay also confirmed that NAT1 significantly inhibited cell proliferation (*P* < 0.05); (**I**–**K**) *In vitro* animal experiments confirmed that overexpression of NAT1 can inhibit the liver metastasis ability of colorectal cancer cells.

We detected NAT1 expression in four colorectal cancer cell lines by qPCR and WB, and found that NAT1 expression was low in HCT116 cell line and high in SW480 cell line. Therefore, HCT116 cell line and SW480 cell line were selected for follow-up experiments (Figure 2A). To manipulate NAT1 expression, we performed overexpression in HCT116 cells (HCT116-NAT1) and NAT1 in SW480 cells (SW480-ShNAT1) was knocked out to establish a stable lentivirus strain. Among the three, sh1 had the best effect. Therefore, we chose sh1 for the follow-up experiment and compared it with overexpression of NAT1. Successful transfection was confirmed by WB and qPCR analysis (Figure 2B, 2C). In the wound scratch assay, the migration area of HCT116-NAT1 cells was significantly smaller than that of HCT116-NC cells, while the migration area of SW480-NC cells was smaller than that of SW480-shNAT1 cells after 24 hours (Figure 2D, 2E). Additionally, the transwell experiment showed that the number of migrated cells was significantly higher in the HCT116-NC group compared to the HCT116-NAT1 group, and similarly, the number of migrated cells was higher in the SW480-shNAT1 group compared to the SW480-NC group (Figure 2F). Moreover, in the plate cloning test, the HCT116-NC group exhibited a significantly higher number of clones compared to the HCT116-NAT1 group. Similarly, the unified SW480-shNAT1 group had a significantly higher number of clones compared to the SW480-NC group (Figure 2G). CK8 test revealed that the proliferation rate of the HCT116-NC group was significantly faster than that of the HCT116-NAT1 group. Similarly, the proliferation rate of the SW480-shNAT1 group was significantly higher than that of the SW480-NC group (Figure 2H).

To further investigate the role of NAT1 in liver metastasis, we established a mouse liver metastasis model of colorectal cancer cells injected into the spleen. The results demonstrated that the NAT1 overexpression group had a significantly lower incidence of liver metastasis and a reduced number of metastatic nodules compared to the control group. Conversely, NAT1 knockdown increased the incidence of liver metastasis (Figure 2I–2K). These findings indicate that NAT1 overexpression significantly inhibits cell proliferation, migration, and metastasis, underscoring its crucial role in liver metastasis of colorectal cancer.

### Overexpression of NAT1 inhibits EMT and glycolysis in colorectal cancer cells

**Figure 3:**
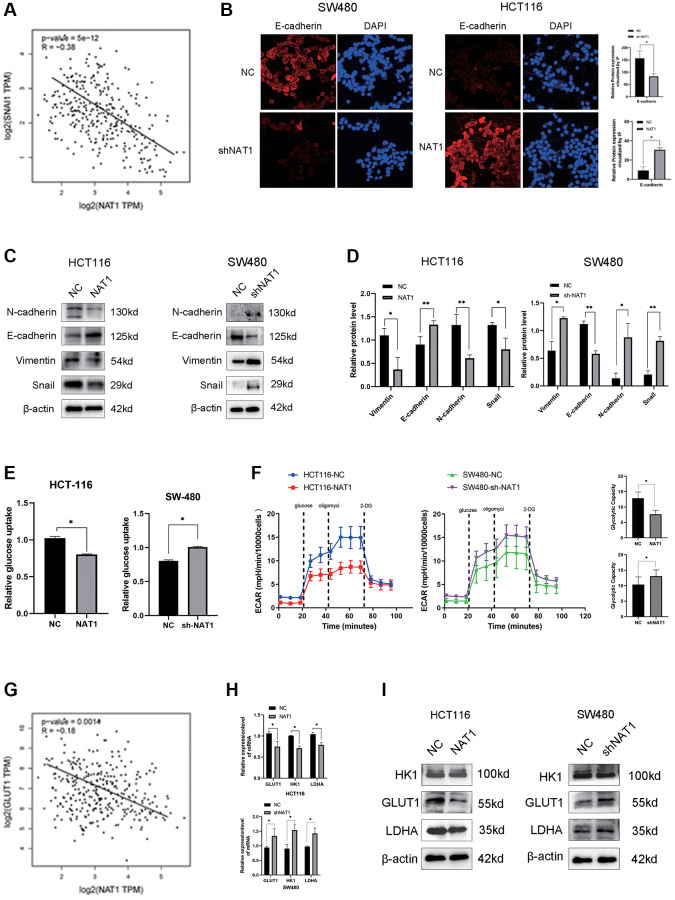
**Overexpression of NAT1 inhibits EMT and glycolysis in colorectal cancer cells.** (**A**) Snail expression in TCGA database was negatively correlated with NAT1; (**B**) Immunofluorescence results showed that overexpression of NAT1 could promote the expression of E-cadherin (*P* < 0.05); (**C**, **D**) Overexpression of NAT1 significantly inhibited the expression of EMT-related proteins (N-cadherin, Snail and Vimentin), and promoted the expression of E-cadherin (*P* < 0.05); (**E**) Overexpression of NAT1 significantly inhibited the glucose uptake ability of colorectal cancer cells; (**F**) ECAR assay showed that NAT1 significantly inhibited the glycolysis and storage capacity of colorectal cancer; (**G**) GLUT1 was negatively correlated with NAT1 expression in TCGA database. (**H**, **I**) Overexpression of NAT1 inhibited the expression of glycolytic related metabolic enzymes (GLUT1, LDHA).

It is well known that EMT plays a key role in tumor metastasis. We speculated whether NAT1 is related to the occurrence and development of EMT. In the TCGA database, we analyzed the expression relationship between NAT1 and the EMT-related protein Snail, showing a significant negative correlation (Figure 3A). In immunofluorescence experiments, E-cadherin of HCT116-NAT1 was significantly increased, while the expression of SW480-shNAT1 was decreased, indicating that NAT1 significantly promoted the expression of E-cadherin (Figure 3B). Next, we also continued to detect the effect of NAT1 on the expression of EMT-related proteins in cells. Overexpression of NAT1 in HCT116 cells reduced levels of EMT-related proteins, including N-cadherin, vimentin, and MMP2 (Figure 3C, 3D). Conversely, overexpression of NAT1 increased E-cadherin levels in CRC cells. The expression of SW480-shNAT1 cells showed the opposite result. Therefore, increased NAT1 levels can inhibit the invasion and migration of colorectal cancer cells by affecting EMT. The results of WB detection were consistent with those of immunofluorescence detection (Figure 3C). In addition, glycolysis is also associated with cancer development and metastasis. We further explored the relationship between NAT1 and glycolysis. First, we detected the relationship between NAT1 and GLUT1 (a key metabolic enzyme in glycolysis) in the TCGA database, and the two were also negatively correlated (Figure 3G), which confirmed our hypothesis. Next, we further analyzed the relationship between NAT1 and glycolysis. Firstly, overexpression of NAT1 significantly inhibited the glucose uptake ability of HCT116 cells, while knockdown of NAT1 significantly promoted the glucose uptake ability of SW480 cells (Figure 3E). Overexpression of NAT1 significantly inhibited the glycolysis capacity of HCT116 cells, while knockdown of NAT1 significantly improved the glycolysis capacity of SW480 cells (Figure 3F). Therefore, we speculated that NAT1 may affect the expression of glycolytic related metabolic enzymes. qPCR and WB results showed that overexpression of NAT1 significantly inhibited the expression of GLUT1, HK1, and LDHA (Figure 3H, 3I). These results indicated that overexpression of NAT1 significantly inhibited EMT and glycolysis of colorectal cancer cells, and further indicated that NAT1 may regulate the occurrence of liver metastasis by regulating EMT and glycolysis of colorectal cancer cells.

### Overexpression of NAT1 blocks PI3K/Akt/mTOR signaling pathway, thereby inhibiting EMT and glycolysis in colorectal cancer cells

**Figure 4:**
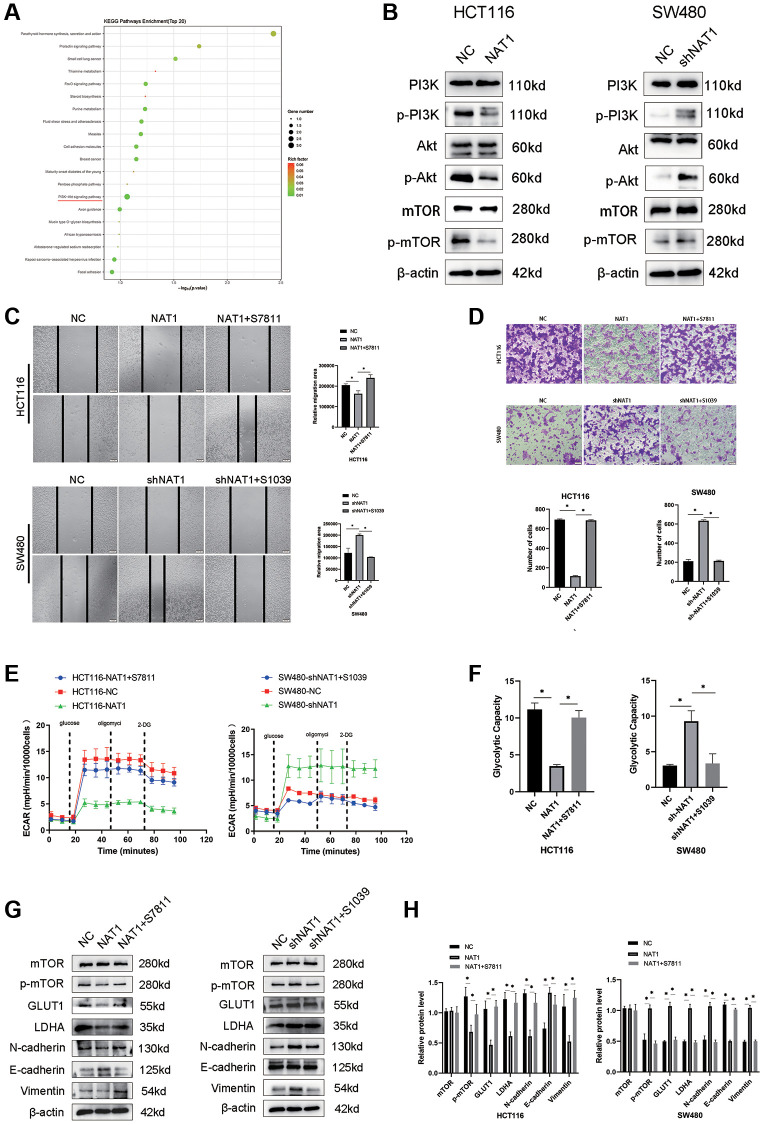
**Overexpression of NAT1 blocks PI3K/Akt/mTOR signaling pathway, thereby inhibiting EMT and glycolysis in colorectal cancer cells.** (**A**) Transcriptome sequencing of showed that PI3K/AKt signaling pathway was enriched; (**B**) Overexpression of NAT1 significantly inhibited the protein expression of PI3K/Akt/mTOR signaling pathway; (**C**, **D**) Reactivation of mTOR can reduce the migration and invasion ability of colorectal cancer cells; (**E**, **F**) Reactivation of mTOR mitigated the inhibition of glycolysis by overexpression of NAT1; (**G**, **H**) Activation of PI3K/Akt/mTOR signaling pathway reversed the inhibitory effect of NAT1 overexpression on EMT (N-cadherin, Vimentin) and glycolytic (GLUT1, LDHA) related proteins.

We performed transcriptome sequencing on the NAT1 overexpressed HCT116 cell line (Figure 4A). The PI3K/AKT/mTOR pathway plays an important role in EMT and glycolysis of tumor cells. Our transcriptomic results showed enrichment in the PI3K/AKT/mTOR pathway, followed by WB experiments to verify that overexpression of NAT1 inhibited the PI3K/AKT/mTOR pathway (Figure 4B). To test whether NAT1 regulates EMT and glycolysis in colorectal cancer cells through the PI3K/AKT/mTOR pathway. Adding mTOR agonist (S7811: MHY1485) to HCT116-NAT1 significantly restored cell migration and glycolysis ability (Figure 4C–4F). The addition of a mTOR inhibitor (S1039: rapamycin) to SW480-shNAT1 cells significantly inhibited cell migration and glycolysis ability (Figure 4C–4F). Moreover, the introduction of the mTOR agonist (MHY1485) to HCT116-NAT1 significantly restored the expression of GLUT1, LDHA, N cadherin, and Vimentin, while promoting the expression of E-cadherin (Figure 4G, 4H). This effect was reversed by adding the mTOR inhibitor (rapamycin) to SW480-shNAT1 cells (Figure 4G, 4H). These results further demonstrate that NAT1 inhibits the EMT and glycolysis ability of colorectal cancer cells through the PI3K/AKT/mTOR signaling pathway, thereby suppressing the proliferation and metastasis of colorectal cancer.

### Overexpression of NAT1 weakens the glycolysis ability of colorectal cancer cells and thus inhibits VEGF expression

**Figure 5:**
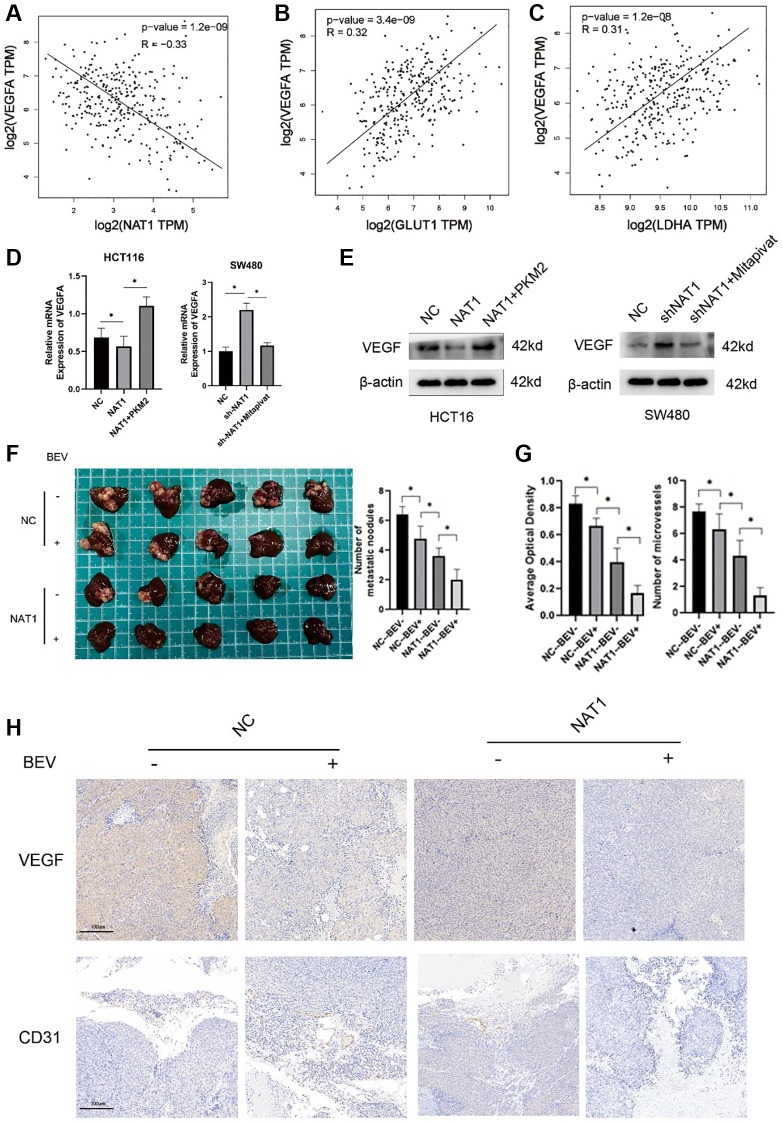
**Overexpression of NAT1 weakens the glycolysis ability of colorectal cancer cells and thus inhibits VEGF expression.** (**A**–**C**) In the TCGA database, VEGFA was negatively correlated with NAT1 expression and positively correlated with the expression of glycolytic related enzymes (GLUT1, LDHA); (**D**, **E**) The glycolysis ability of colorectal cancer cells is correlated with the expression of VEGF, and glycolysis can enhance the expression of VEGF; (**F**) *In vitro* animal experiments showed that overexpression of NAT1 combined with VEGF inhibitors could better inhibit the occurrence of liver metastasis; (**G**, **H**) Overexpression of NAT1 combined with VEGF inhibitors can better reduce the expression of VEGF and CD31 in liver metastases.

In the TCGA database, VEGF expression was negatively correlated with NAT1 expression, but positively correlated with GLUT1 and LDHA (Figure 5A–5C). Therefore, we investigated the effect of glycolysis on VEGF expression in colorectal cancer cells by adding shikolin, an effective and specific pyruvate kinase M2 (PKM2) inhibitor, to SW480-shNAT1 cells. In contrast, the addition of Mitapivat (pyruvate kinase PKM2 activator) to HCT116-NAT1 cells significantly increased VEGF expression. This further verified that NAT1 inhibits VEGF expression by regulating the glycolysis ability of colorectal cancer cells (Figure 5D, 5E). In addition, overexpression of NAT1 in combination with antiangiopoietin (bevacizumab) was more effective in preventing tumor metastasis in animal studies (Figure 5F). Similarly, in the immunohistochemical results of metastatic lesions, overexpression of NAT1 combined with bevacizumab significantly inhibited the expression of VEGF and CD31 (Figure 5G, 5H). In immunohistochemistry, CD31 is primarily used to demonstrate the presence of endothelial cell tissue and is used to assess tumor angiogenesis, which may indicate the extent of a rapidly growing tumor. The results indicated that the combination of overexpression of NAT1 and bevacizumab significantly inhibits the proliferation of blood vessels in the metastatic lesions, thus reducing the occurrence and development of liver metastasis.

## DISCUSSION

Human aromatic amine N-acetyltransferase 1 (NAT1) has traditionally been characterized as a phase II heterometabolic enzyme involved in the inactivation and bioactivation of certain environmental aromatic amine carcinogens, utilizing acetyl-CoA [24–26]. The expression of NAT1 varies among individuals and is typically reduced in various cancers, including estrogen receptor-positive (ER+) breast cancer and colorectal cancer. Its low expression is strongly linked to cancer development and metastasis [22, 27, 28]. While NAT1 expression is generally low in colorectal cancer, its role in liver metastasis of colorectal cancer has not been extensively studied. Our findings indicate that patients with liver metastasis exhibit lower NAT1 expression levels, which motivates us to further investigate the underlying mechanism of action. CRC is a prevalent malignant tumor in humans, and there is an urgent need to further comprehend its molecular mechanism [29]. The objective of this study was to meticulously investigate the role of NAT1 in CRC development and identify the associated signaling mechanisms. EMT is a physiological process that enhances cell invasion and migration, and it has been recognized as a crucial factor in tumor metastasis and the progression of various cancers [7]. The expression levels of specific molecular markers can reflect the extent of EMT. Notably, reduced expression of E-cadherin, along with upregulated expression of N-cadherin and vimentin, significantly contribute to the occurrence of EMT [30, 31]. Akt (protein kinase B) is an important downstream effector of the PI3K/Akt/mTOR pathway. It can directly or indirectly affect EMT-related transcription factors, intercellular adhesion molecules, matrix metalloproteinases and other factors that regulate EMT. By regulating these factors, the PI3K/Akt/mTOR pathway can promote or inhibit the EMT process of cells, thereby affecting tumor metastasis and invasion. Our findings indicate that NAT1 inhibits EMT progression by upregulating E-cadherin and downregulating N-cadherin and vimentin expression. These results imply that NAT1 may regulate liver metastasis in colorectal cancer by inhibiting the progression of EMT in colorectal cancer.

One prominent metabolic phenotype observed in cancer cells is aerobic glycolysis, commonly known as the Warburg effect [32], the Warburg effect is a common metabolic reprogramming phenomenon, particularly associated with cancer cells. It refers to the fact that even when oxygen is plentiful, cancer cells tend to produce energy through a process called lactic acid fermentation, rather than the oxidative phosphorylation process normally used by normal cells. This phenomenon causes cancer cells to churn out lactic acid and generally require more glucose. The Warburg effect allows cancer cells to adapt to the high energy and biomolecular requirements required for rapid proliferation. Growing evidence suggests that glycolysis is not only driven by anoxic conditions but also by carcinogenic signals, including MAPK, PI3K/AKT [33], and WNT/β-catenin pathways [34, 35]. In the case of hepatocellular carcinoma (HCC), previous studies have demonstrated that the Warburg effect contributes to its proliferation and progression. Several carcinogenic signaling pathways, such as PI3K/AKT, JNK1, HIF-1α, and c-MYC, are involved in the regulation of aerobic glycolysis in HCC [36–42]. However, the role and molecular mechanism of aerobic glycolysis in liver metastasis of colorectal cancer remain unclear. NAT1 is a metabolic enzyme that has been investigated in relation to breast cancer energy metabolism. Studies have shown that NAT1 inhibits the glycolytic capacity of breast cancer cells, thus suppressing their occurrence, development, and metastasis [43]. Based on this, we hypothesized that NAT1 might regulate liver metastasis by modulating the glycolytic capacity of colorectal cancer cells. Experimental results indicated that NAT1 inhibited the glycolysis of colorectal cancer cells, consequently inhibiting the occurrence of liver metastasis. RNA sequencing data suggests that NAT1 may regulate liver metastasis of colorectal cancer through the PI3K/AKT/mTOR signaling pathway and inhibits the expression of glycolytic-related metabolic enzymes such as GLUT1 and LDHA. According to our transcriptome sequencing results, the PI3K/AKT/mTOR pathway is significantly affected, and previous studies have shown that the PI3K/AKT/mTOR pathway is correlated with cancer and glycolysis. After Akt is activated, it will promote glucose uptake and activation of glycolytic enzymes, thereby increasing cells’ utilization of glucose. In addition, mTOR complex 1 is also involved in regulating the glycolysis pathway, affecting the utilization and metabolism of glucose by cells by directly affecting post-translational modifications of glycolysis-related enzymes or indirectly regulating the activity of transcription factors. Of course, in addition to the PI3K/AKT/mTOR pathway, other pathways may also exist in NAT1 interactions to regulate colorectal cancer glycolysis. This should be further clarified in future studies.

The occurrence, proliferation, and metastasis of tumors are closely associated with abundant neovascularization in the tumor microenvironment [44]. Endothelial cells primarily rely on aerobic glycolysis to drive angiogenesis, which is frequently observed during this process [45]. Approximately 85% of adenosine triphosphate (ATP) production in endothelial cells (ECs) depends on aerobic glycolysis rather than oxidative phosphorylation, particularly during angiogenesis. This preference for glycolysis is due to its ability to generate ATP at a faster rate compared to oxidative metabolism, which enables ECs to meet the high energy demands associated with dynamic growth and division [13, 14]. Recent studies have demonstrated a two-fold increase in the glycolysis rate when quiescent endothelial cells transition to rapid vascular sprouting [13]. VEGF, an important angiogenic factor in tumors, is involved in the initial stages of tumor initiation, progression, and metastasis [11]. Lactic acid, a byproduct of metabolic glycolysis, is an active metabolite that accumulates at sites of inflammation and regulates cellular signaling [46–48]. Lactic acid can induce the expression of VEGF, thereby promoting angiogenesis [49]. Our findings indicate that overexpressing NAT1 in colorectal cancer cells can inhibit the glycolytic capacity of these cells, consequently suppressing the expression of VEGF and affecting the formation of liver metastases. Furthermore, animal experiments suggest that simultaneous overexpression of NAT1 and the application of angiopoietin inhibitors yield more pronounced effects, significantly preventing the occurrence of liver metastasis in tumors. Immunohistochemical analysis of mouse liver tissue also supports this observation, as highly metastatic lesions exhibit higher VEGF expression and increased neovascularization.

**Figure 6:**
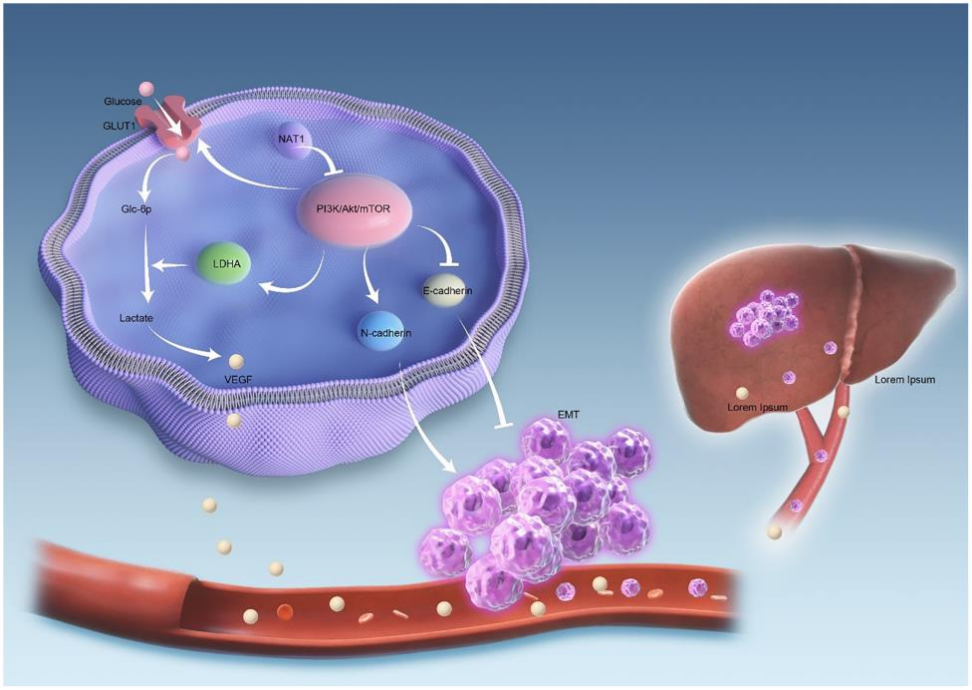
**The mechanism diagram shows that NAT1 inhibits liver metastasis of colorectal cancer.** Overexpression of NAT1 inhibits PI3K/AKT/mTOR signaling pathway. On the one hand, overexpression of NAT1 inhibited the occurrence and development of EMT in tumor cells. On the other hand, NAT1 weakens the glycolytic ability of colorectal cancer cells by inhibiting the expression of GLUT1 and LDHA. The decreased glycolytic ability leads to the decreased expression of VEGF in colorectal cancer cells, and the decreased expression of VEGF leads to the decrease of neovascularization and vascular permeability in liver metastases, thus reducing the occurrence of liver metastasis.

Figure 6 illustrates the mechanism investigated in this study. The overexpression of NAT1 is shown to inhibit the PI3K/AKT/mTOR signaling pathway. Firstly, NAT1 overexpression inhibits the occurrence and progression of epithelial-mesenchymal transition (EMT) in tumor cells. Additionally, NAT1 weakens the glycolytic ability of colorectal cancer cells by suppressing the expression of GLUT1 and LDHA. This reduced glycolytic capacity leads to decreased expression of VEGF in colorectal cancer cells, which in turn reduces neovascularization and vascular permeability in liver metastases, ultimately resulting in a decreased occurrence of liver metastasis. Therefore, it can be inferred that NAT1 suppresses liver metastasis of colorectal cancer by modulating EMT and glycolysis in colorectal cancer cells through the PI3K/AKT/mTOR signaling pathway (Figure 6).

In conclusion, the results demonstrate that NAT1 plays a significant role in inhibiting the epithelial-mesenchymal transition (EMT) of colorectal cancer. Additionally, it effectively suppresses the glycolysis ability and reduces VEGF expression in colorectal cancer cells by modulating the PI3K/AKT/mTOR signaling pathway. These combined effects effectively hinder the occurrence of liver metastasis in colorectal cancer, thus offering a promising therapeutic target for clinical interventions.

## MATERIALS AND METHODS

### Patient specimens

From June 2016 to September 2022, pathological sections of colorectal cancer and colorectal cancer with liver metastasis were obtained from the Department of Pathology at the First Affiliated Hospital of Anhui Medical University to determine protein levels. The study also involved collecting and investigating the basic information of all patients, who provided informed consent. Ethical approval for this study was obtained from the ethics committee of the First Affiliated Hospital of Anhui Medical University; ethical number of animal experiments: LLSC20221082.

### Cell culture and transfection

The human CRC cell lines (SW480 (BTCC-1181), LOVO (SNL-070), HCT116 (SNL-077), and DLD-1 (SNL-076)) were obtained from Wuhan Punosai Life Technology Co., Ltd., China. All cells were cultured in DMEM (Sparkjade, Shandong, China) supplemented with 10% fetal bovine serum (FBS) (ExCell Bio, Shanghai, China) under standard conditions of 37°C, 5% CO_2_, and humidification. The overexpression of NAT1 and knockdown of NAT1 (LV3-NAT1-HOMO-93: GATCCGAGCTGTTCCCTTTGA; LV3 NAT1 - homo - 197: GATGGTGTCTCCAGGTCAATC; Lentiviral vector LV3-NAT1-HOMO-405: GGAGTTAATTTCTGGGAAGGA) and its negative control pLVXshRNA1 (SH-NC) were purchased from Gima gene (Shanghai, China). HCT116 and SW480 cells were transfected with Polybrene following the manufacturer's protocol. The cell density should reach 80–90% before transfection, and 1 microliter (μl) of lentiviral particles should be added to the cell medium. The petri dishes were returned to the cell incubator and incubated at 37°C and 5% CO_2_. Ensure that the cells are kept at the appropriate temperature and CO_2_ concentration during transfection. After 24 h of infection, puromycin was added to the cells to select for resistant cells. The knockdown efficiency was evaluated by western blotting (WB) and RT-qPCR.

### RNA extraction and reverse transcription-quantitative polymerase chain reaction (RT-qPCR)

We utilized Trizol reagent (Sparkjade, China) for the extraction of total RNA from cultured cells. Quantitative RT-PCR was performed using the PrimeScript RT Kit (Accurate, Hunan, China) and SYBR Premix Ex Taq (Accurate, Hunan, China). The following primer sequences were employed: NAT1: Forward 5′-GGTGTCTCCAGGTCAATCATCTTCT-3′; Reverse 5′-CGACAATGTAGTTCCTGCCATCAAT-3′; GLUT1: Forward 5′-GCTTCCAGTATGTGGAGCAAC-3′; Reverse 5′-CCTGTGCTCCTGAGAGATCC-3′; HK1: Forward 5′-GAGTCTGGACGCGGGAATC-3′; Reverse 5′-CAGGTGGGCTCCTCATAAGC-3′; LDHA: Forward 5′-GCAGGTGGTTGAGAGTGCTTA-3′; Reverse 5′-CTTCAAACGGGCCTCTTCCT-3′; VEGF: Forward 5′-ATGAACTTTCTGCTCTCTTGGGTACA-3′; Reverse 5′-GCAGATGTGACAAGCCAAGGCGGTG-3′.

The qPCR conditions were as follows: pre-denaturation at 95°C for 30 seconds, followed by 40 cycles of denaturation at 95°C for 5 seconds, annealing and extension at 60°C for 30 seconds. GAPDH was used as the internal reference. The relative mRNA expression of the target genes was determined using the 2^−ΔΔCq^ method.

### Immunohistochemistry (IHC) and immunofluorescence (IF)

The tissue wax blocks were obtained from the pathology department, and tissue sections were prepared. Each slide was coated with a rabbit polyclonal antibody against NAT1 (diluted 1:300; Wuhan, China). The slides were then incubated overnight at 4°C, followed by three washes with PBS. Subsequently, the corresponding biotinylated secondary antibody (Shanghai Dako, China) was applied and incubated for 30 minutes. To visualize the peroxidase reaction, 3,3′-diaminobenzidine (DAB) (Shanghai Dako, China) was administered, and the tissue sections were counterstained with hematoxylin. The immunohistochemistry (IHC) strength scores were assigned as follows: 0 for no staining, 1 for slight staining, 2 for moderate staining, and 3 for strong staining.

If performing staining, the cells were cultured on 12-well plates with cell climbing sheets, fixed with 4% paraformaldehyde, treated with 0.5% Triton X-100 (Sparkjade, China), blocked with 1% goat serum albumin, and incubated overnight at 4°C with the primary antibody e-cadherin (20874-1-ap, 1:100). Subsequently, the cells were treated with a secondary antibody, Goat anti-rabbit IgG H&L (Alexa Fluor 594). Nuclei were counterstained with DAPI, which contained an anti-fluorescence quenching agent (Sparkjade, China). Finally, the images were captured using a laser confocal microscope.

### Cell proliferation assay

Cell proliferation was assessed using the cell counting kit-8 (CCK-8) assay. Briefly, stably transfected HCT116 and SW480 cells were seeded into 96-well plates at a density of 2000 cells per well and incubated at 37°C in a 5% CO_2_ environment. At 0, 24, 48, and 72 hours, CCK-8 reagent was added to each well. The absorbance at 450 nm was measured using a Bio-Rad Laboratories, Inc. (Hercules, CA, USA), spectrophotometer.

For the colony formation assay, HCT116 and SW480 cells were seeded into 6-well plates at a density of 1000 cells per well and cultured for two weeks. After the incubation period, the culture medium was aspirated, and the cells were washed three times with PBS. Next, the cells were fixed with 4% paraformaldehyde for 30 minutes, followed by three washes with PBS. Subsequently, the cells were stained with 1% crystal violet for 20 minutes and washed three times with PBS. Finally, the number of colonies was observed and counted.

### Cell migration and invasion assays

Cell migration and invasion were tested in the Transwell Chamber (Corning, Inc., Corning, NY, USA). In migration experiments, stably transfected cell lines (HCT116-NAT1 and SW480-shNAT1) were digested with trypsin and suspended in DMEM medium (2 × 10^5^ cells/mL). In the upper chamber, cells were inoculated with a medium containing 1% fetal bovine serum, and in the lower chamber, cells were filled with a medium containing 20% fetal bovine serum. After the cells on the surface of the membrane were removed with cotton swabs, the cells were incubated until the specified time, and the penetrating cells were fixed in methanol and stained with Giemsa for 30 min. The cells that migrated or invaded were counted in five fields under a 100x magnifying glass.

### Western blot (WB) analysis

Proteins were extracted from stably transfected HCT116 and SW480 cells and subjected to sodium dodecyl sulfate polyacrylamide gel electrophoresis (SDS-PAGE) for separation. The separated proteins were then transferred onto a polyvinylidene fluoride (PVDF) membrane. Subsequently, the membrane was blocked with rapid blocking solution for ten minutes, followed by incubation with primary antibodies overnight at 4°C. After that, the membrane was incubated at 37°C for one hour with either Goat anti-rabbit or anti-mouse secondary antibody (1:5000; Zen Bioscience, Chengdu, China). Visualization of the membrane was performed using the Bio-Rad gel imaging system (Bio-Rad, Hercules, CA, USA) with the WB Kit (Advansta, Menlo Park, CA, USA). ImageJ software (version 6.0; National Institutes of Health, Bethesda, MD, USA) was utilized for result analysis, with β-actin serving as the internal control. The primary antibodies used for WB analysis were as follows: Zen-Bioscience (China): β-actin (60004-1-Ig, 1:5000); Proteintech Group, Inc. (China): E-cadherin (20874-1-AP, 1:1000), Wnt1 (27935-1-AP, 1:1000); Abcam (UK): N-cadherin (ab76001, 1:1000), NAT1 (ab109114, 1:1000), Vimentin (ab9254, 1:1000), MMP2 (ab92536, 1:1000); Abmart (China): Bax (Abmart, 32503, 1:1000), Bcl-2 (ab32124, 1:1000), Cleaved caspase-3 (P42574, 1:1000), Cleaved PARP (P09874, 1:3000), GLUT1 (21829-1-AP, 1:1000), HK1 (15656-1-AP, 1:1000), LDHA (19987-1-AP, 1:1000), mTOR (66888-1-Ig, 1:1000), p-mTOR (67778-1-Ig, 1:1000), PI3K (20584-1-AP, 1:1000), Akt (60203-2-Ig, 1:1000), p-Akt (66444-1-Ig, 1:1000).

### Seahorse XF glycolysis stress test

The day prior to the test, the Seahorse XFe/XF analyzer should be turned on and allowed to reach a stable temperature. Cells should be inoculated into Seahorse XF microplates at a predetermined density using an appropriate cell growth medium. Additionally, a sensor probe plate should be hydrated overnight in a 37°C CO_2_-free incubator using Seahorse XF calibration solution. On the day of the experiment, the test solution is prepared by adding an additive to the Seahorse XF base medium. Agilent Seahorse recommends starting with 2 mmol/L glutamine; however, the specific medium components required may vary depending on the cell type or *in vitro* culture conditions. The test solution should be heated to 37°C and its pH adjusted to 7.4 using 0.1N NaOH (Note: Agilent Seahorse recommends filtering the solution after pH adjustment to remove bacteria). The solution should be kept at 37°C until it is ready for use. The cell culture microplates should be taken out from a 37°C CO_2_ incubator, and the cells should be examined under a microscope to confirm confluence. The test solution should be removed from the water bath. Using a multi-channel pipette, the warm test solution replaces the cell growth medium, and the cell culture microplates are then placed in a 37°C CO_2_-free incubator for 45 minutes to 1 hour. Finally, the Seahorse XF glycolysis stress test can be run.

### Animal experiment

Establishment of nude mouse (D000521, BALB/c-Nude, (Foxn1^nu^) mut/mut) spleen injection liver metastasis model: preparation tools and materials: anesthetic, disinfectant, gloves, syringe, mouse colorectal cancer cell suspension. Select four-week-old male nude mice, and ensure that they are in good health, and divide them into two groups of 6 each. The mice were anesthetized with ether to ensure that the mice were in a pain-free state. Anesthesia by inhalation of ether. Put 5 ~ 10 mL defibrated cotton soaked in ether at the bottom of the glass container, then put the animal in, cover the lid, and the animal enters the anesthesia state for 20 ~ 30 seconds. Place the mouse on a sterile operating bench and perform necessary disinfection steps, including sterile shaving and skin disinfection. Load the pre-prepared mouse colorectal cancer cell suspension in the syringe (inject 2 × 10^6^ cells per mouse). Use a scalpel to make a small incision in the spleen area of the mouse, slowly insert the syringe into the spleen, and inject an appropriate amount of colorectal cancer cell suspension. After the injection is complete, the incision is closed with sutures to ensure good wound healing. Place the mouse in a warm, comfortable recovery box to ensure recovery and maintain a stable body temperature. Mice were sacrificed four weeks after injection and liver metastases were examined.

Figure 2J animal experiments were divided into four groups of animal models: HCT116-NC, HCT116-NAT1, SW480-NC, and SW480-shNAT1. Each mouse in each group was injected with 2 × 10^6^ cells into the spleen of each mouse. The mice were killed four weeks later, and the liver tissues were taken out.

Figure 5F animal experiments were divided into four groups: HCT116-NC, HCT116-NC+bevacizumab, HCT116-NAT1 and HCT116-NAT1+bevacizumab. Each mouse in each group was injected with 2 × 10^6^ cells in the spleen of each mouse.

Intraperitoneal injection of bevacizumab: one week after the completion of the transfer model, the mice were randomly divided into four groups (5 mice in each group), and 5 mg/kg bevacizumab was injected intraperitoneally; the control group was injected intraperitoneally with PBS, treated eight times. The mice were sacrificed at four weeks, and the liver metastases were observed. All experimental operations follow the laboratory’s ethics and experimental operation guidelines.

Mice are placed in IVC cages, and the density of animals should not be too large. Do not inject CO_2_ into the euthanasia box before placing the animal. Put the animal into the cage, close the lid, connect the CO_2_ pipe at the entrance where the water bottle is placed, open the valve door of the gas bottle, and replace the volume of the euthanasia box 10%–30% of the rate of CO_2_ into the box every minute, so that CO_2_ is full of the cage. After the mice fainted and lost the ability to exercise, the gas flow could be increased, and the maximum flow could not exceed 0.5 KPa. After confirming that the mice did not move, did not breathe, the pupils dilated, CO_2_ was turned off, and the mice were observed for two minutes to determine death.

### Mouse imaging experiment *in vivo*

The target cells of logarithmic growth stage were taken and inoculated into a 6-well plate. The cells could be transfected when the cell fusion degree reached 80%-90% in the well culture plate. During transfection, the target cells, liposome transfection reagents and sufficient fluorescent plasmid carrier suspension were co-cultured for 6 h, and then fresh culture medium was added. 48 h after transfection, the cells were digested with pancreatic enzyme and inoculated into 6-well plates at a ratio of 1:6 with the addition of antibiotic G418, then the medium was changed every 2 d and G418 screening was maintained until single-cell resistant clones emerged. The mice were injected with labeled fluorescent cells for four weeks. Isoflurane ensured that the mice were completely anesthetized to mitigate image blurring caused by motion. Turn on the Aura device and ensure that the light source, filter, and other imaging parameters have been adjusted to the appropriate settings. The anesthetized mice were placed on the imaging table and the Aura device was activated to begin image acquisition.

### Statistical method

Data are expressed as the mean ± SD of at least three independent experiments. Graph generation and statistical analysis were done using GraphPad Prism 9 (GraphPad Software). The student *T*-test with or without Welch correction and ANOVA with post hoc Dunnet test were used to compare the mean values of two or more groups, respectively. The expression difference of NAT1 in normal tissues and tumor tissues was analyzed by Mann-Whitney *U*-test and Chi-square test to analyze the transcriptome data and IHC staining score. A probability (*P*) value < 0.05 was considered statistically significant.

### Availability of data and materials

The data underlying this article are available in the article and in its online Supplementary Material.
